# Supplementary material for: Search-and-remove genome editing allows selection of cells by DNA sequence
Source: Nat Commun. 2025 Dec 8;16:10985. doi: 10.1038/s41467-025-66896-1 (PMC12689631; doi:10.1038/s41467-025-66896-1)
Supplement: Supplementary file 1 — Supplementary Information [file 41467_2025_66896_MOESM1_ESM.pdf]

Supplementary Information

Search-and-remove genome editing allows selection of cells by DNA sequence

Luise Fast et al.

Supplementary Figures

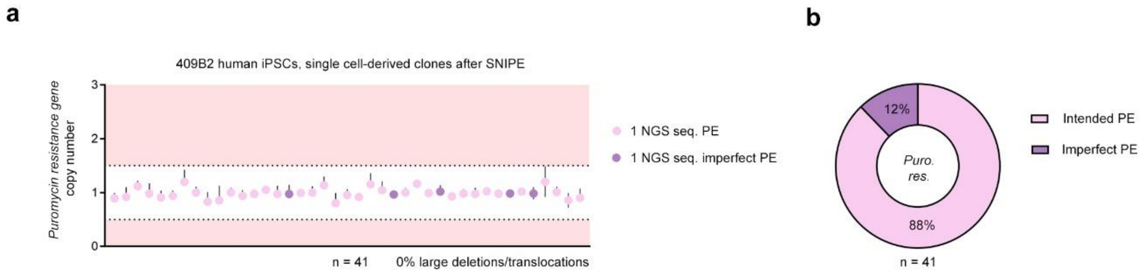

Supplementary Figure 1: Genotype and copy number analysis of single cell-derived clones after SNIPE of puromycin resistance gene repair. (**a**) Target site sequencing and droplet digital (dd) PCR copy number analysis of cellular clones after repairing the puromycin resistance gene using prime editing (PE) in human 409B2 iPSCs subsequent SNIPE. The copy number of target sequences relative to the gene *FOXP2* in cellular clones is plotted as a filled circle. The puromycin resistance gene has a copy number of one (monoallelic). The circles are in rose and purple to represent genotypes with the intended substitution (PE), or imperfect PE (PE and additional indels or substitution). The measure of center for the error bars represents the ratio of the Poisson-corrected number of target to reference molecules. The error bars represent the 95% confidence interval of this measurement. The numbers of cellular clones analyzed is stated and none has copy number loss. (**b**) A pie chart gives the percentage of genotypes of the cellular clones from a.

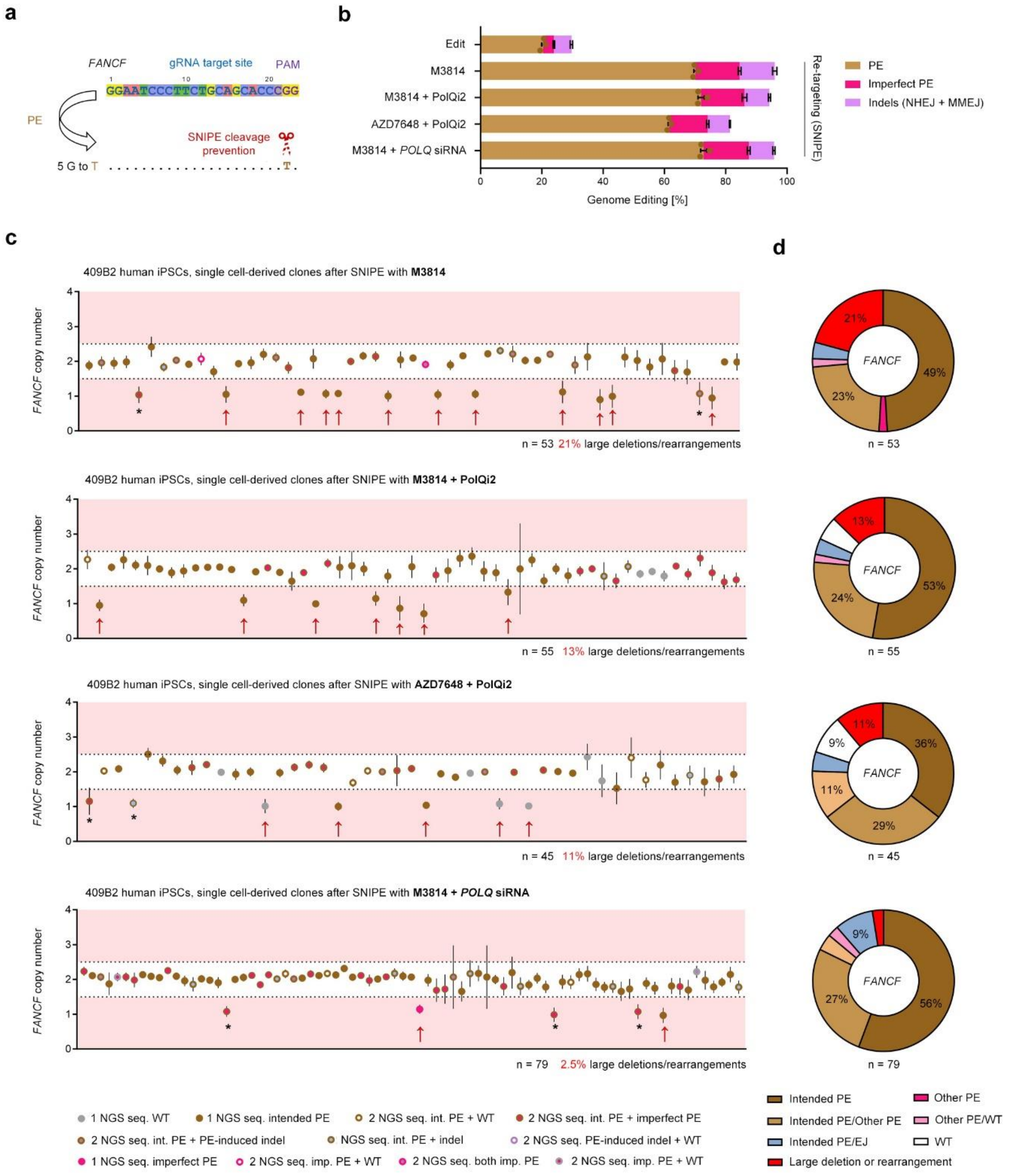

Supplementary Figure 2: SNIPE efficiencies and copy number analysis of single cell-derived clones for different end-joining repair substance combinations. (**a**) Target site sequence of the gRNA used for SNIPE in the *FANCF* gene. The intended mutation introduced by prime editing (PE), which prevents SNIPE cleavage, is also shown. (**b**) Genome editing efficiencies for PE of human 409B2 iPrime stem cells of *FANCF*, and after subsequent SNIPE (with repair pathway inhibition). M3814 and AZD7648 are small molecule inhibitors of DNA-PKcs and thus NHEJ. PolQi2 is a small molecule inhibitor of Polϴ and thus MMEJ. PolQ siRNA targets the mRNA of the gene *POLQ* that encodes for Polϴ. PE is indicated in brown, imperfect PE in light brown, and indels in magenta. Independent biological replicates were performed (n =3) and are depicted as dots. Error bars show the s.e.m. (**c**) Target site sequencing and droplet digital (dd) PCR copy number analysis of cellular clones after CRISPR cleavage and end-joining pathway inhibition. The copy number of target sequences relative to the gene *FOXP2* in cellular clones is plotted as a filled or open circle when one predominant DNA

sequence (seq.) (apparent homozygous) or two DNA sequences with a similar frequency (apparent heterozygous) were obtained, respectively. The circles are in different color shades to represent different combinations of unmodified (WT, wild type) chromosomes, chromosomes carrying intended substitutions (PE), intended substitution and additional indels (imperfect PE), only indels (indel), or indel types that were already present after the initial editing and are unlikely introduced by SNIPE (PE-induced indel). Red arrows point to clones with copy number changes. Black asterisks indicate an indel at the ddPCR primer/probe site that results in inability to amplify this locus for one chromosome. The measure of center for the error bars represents the ratio of the Poisson-corrected number of target to reference molecules. The error bars represent the 95% confidence interval of this measurement. The numbers of cellular clones analyzed is and percentage of those with copy number change are stated. (**d**) A pie chart gives the percentage of genotypes of the cellular clones from c. Prime editing outcomes are differentiated between intended PE and any other PE that includes imperfect PE and PE-induced indels. Other indels are likely due to end-joining repair (EJ).

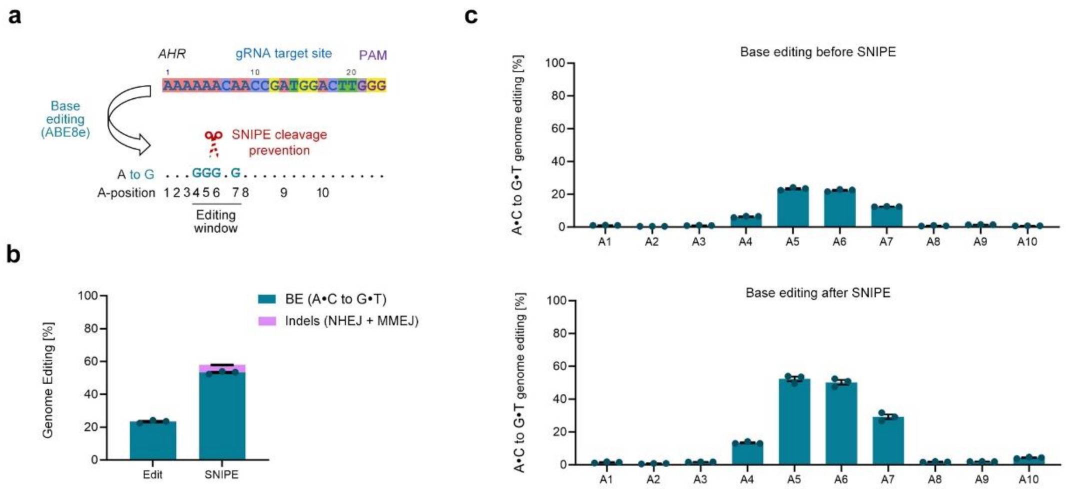

Supplementary Figure 3: SNIPE efficiency after base editing. (**a**) Target site sequence of the gRNA used for base editing and SNIPE at the *AHR* target. The positions of intended A to G mutations introduced by the deaminase, which prevent SNIPE cleavage, are also shown and labelled A1 to A10. (**b**) Genome editing efficiencies for base editing (BE) using recombinant adenine base editor ABE8e in human 409B2 iPSCs before and after SNIPE. Base editing (A to G substitution) is indicated in teal, and indels in magenta. Independent biological replicates were performed (n =3) and are depicted as dots. Error bars show the s.e.m. (**c**) Position dependent (A1 to A10) efficiency of A to G substitutions before and after SNIPE related to b.

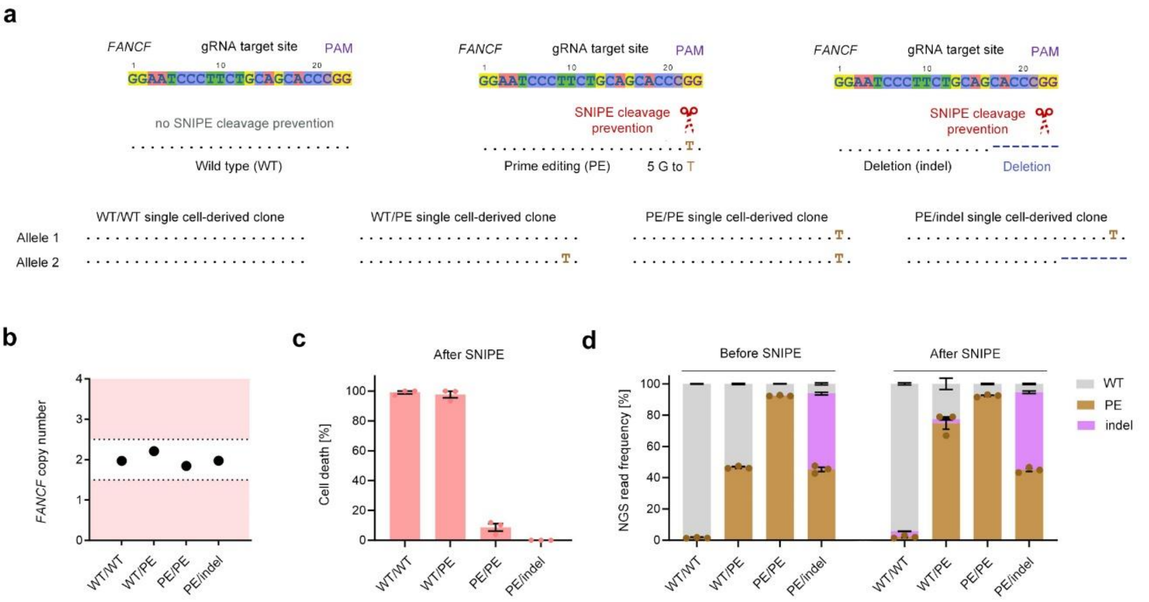

Supplementary Figure 4: SNIPE efficiency and cell death of single cell-derived clones with monoallelic or biallelic editing. (**a**) Genotypes of both alleles at the *FANCF* target site of single cell-derived clones used for SNIPE: WT/WT (wild type), PE/PE, PE/WT, and PE/indel. The cell mix before single cell generation underwent Prime editing (PE) to install the *FANCF* +5 G to T mutation. Both the PE edit and the indel destroy the gRNA target site and prevent SNIPE cleavage of the gene. The deletion in the PE/indel clone is indicated by a dash. (**b**) Target site sequencing and droplet digital (dd) PCR copy number analysis of human 409B2 iPrime stem cell clones from a. The copy number of target sequences relative to the gene *FOXP2* in cellular clones is plotted. The measure of center for the error bars represents the ratio of the Poisson-corrected number of target to reference molecules. (**c**) Cell death after selection by SNIPE by a resazurin assay. (**d**) NGS read frequency for mock and SNIPE edit. The PE allele is colored in brown, wild type in grey, and indels in light purple. Independent biological replicates were performed (n = 3 for c and d) and are depicted as dots for the PE sequence reads and cell survival. Error bars show the s.e.m.

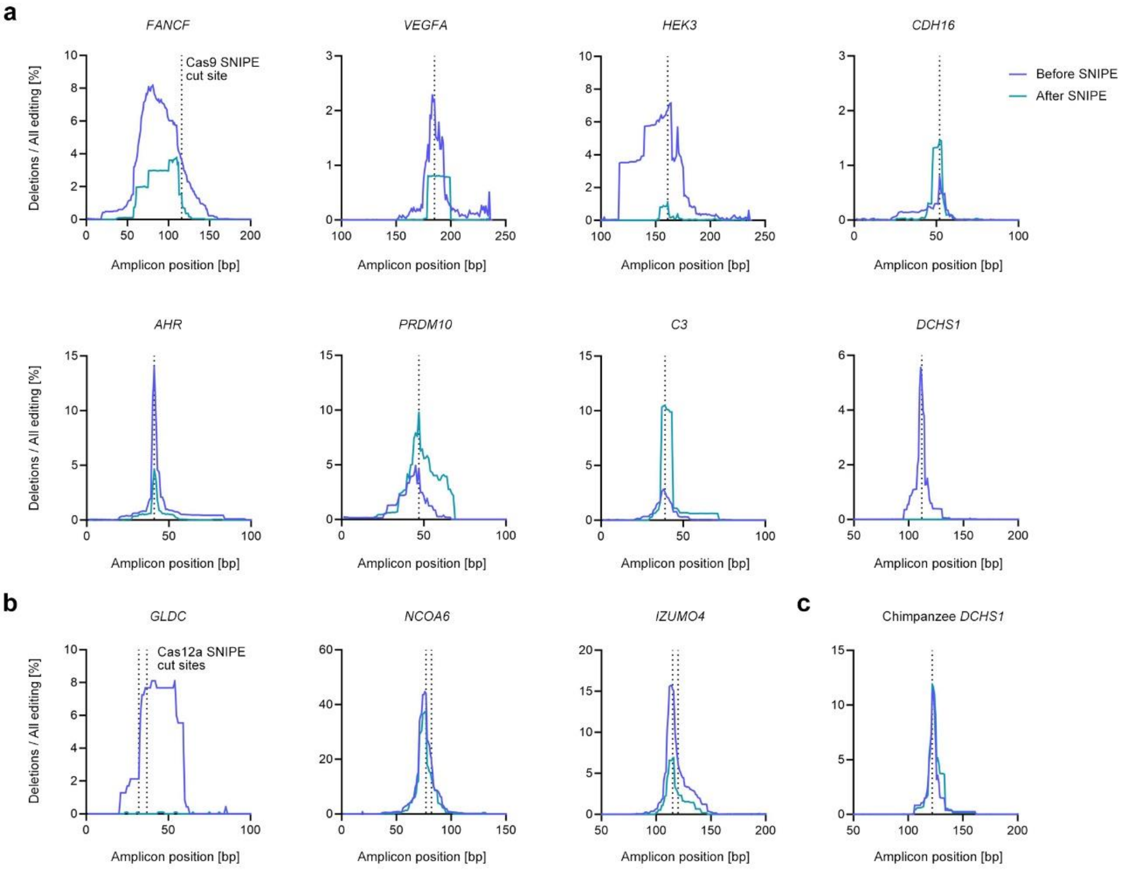

Supplementary Figure 5: Deletion patterns before and after SNIPE. (**a**) Deletion pattern shapes before (purple) and after (turquoise) SNIPE with Cas9 RNP (Cas9-Hifi) for editing in human 409B2 hiPSCs (corresponding to Fig. 2a-c). (**b**) Deletion pattern shapes before and after SNIPE with Cas12a RNP (Cpf1-Ultra) for editing in human hiPSCs (corresponding to Fig. 2d). (**c**) Deletion pattern shapes before and after SNIPE with Cas9 RNP (Cas9-Hifi) for editing in Sandra A chimpanzee hiPSCs (corresponding to Fig. 2d). Each line is the mean of independent biological replicates (n = 3). The vertical dotted lines indicate the position of the cleavage sites.

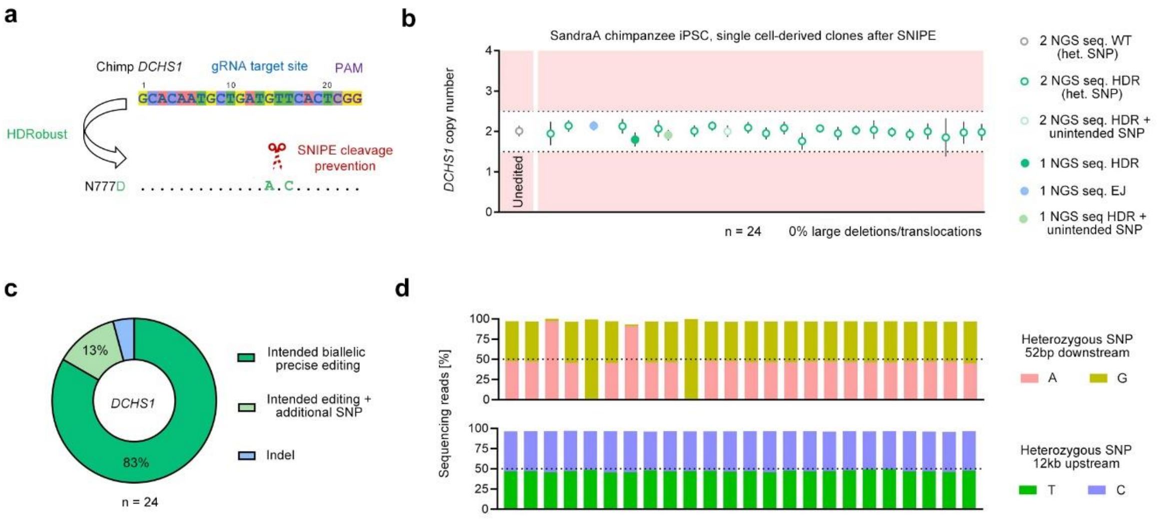

Supplementary Figure 6: Single cell-derived clones after editing with SNIPE in chimpanzee iPSCs. (**a**) Target site sequence of the gRNA used for editing and SNIPE in the DCHS1 gene. The intended mutations introduced by an exogenous DNA donor, which prevent SNIPE cleavage, are also shown. (**b**) Target site sequencing and droplet digital (dd) PCR copy number analysis of cellular clones after editing *DCHS1* in Sandra A chimpanzee iPSCs using HDRobust and subsequent SNIPE. The copy number of target sequences relative to the gene *FOXP2* in cellular clones is plotted as a filled or open circle when one predominant DNA sequence (seq.) (apparent homozygous) or two DNA sequences with a similar frequency (apparent heterozygous) were obtained, respectively. The circles are in shades of green and blue to represent different combinations of unmodified (WT, wild type) chromosomes, chromosomes modified by HDR and chromosomes modified by NHEJ or MMEJ (summarized as end joining, EJ). Incorporation of the targeted substitution is quantified as HDR and observations of unintended SNP edits are stated. The sequencing amplicon contains a normally heterozygous SNP in addition to the intended edit, which should give two different DNA sequences. The measure of center for the error bars represents the ratio of the Poisson-corrected number of target to reference molecules multiplied by two for the diploid state of the reference gene. The error bars represent the 95% confidence interval of this measurement. The numbers of cellular clones analyzed and none has copy number loss. (**c**) A pie chart gives the percentage of genotypes of the cellular clones from b. (**d**) Genotypes of SNPs upstream (52bp) and downstream (12kb) of the target site from the cellular clones in b.

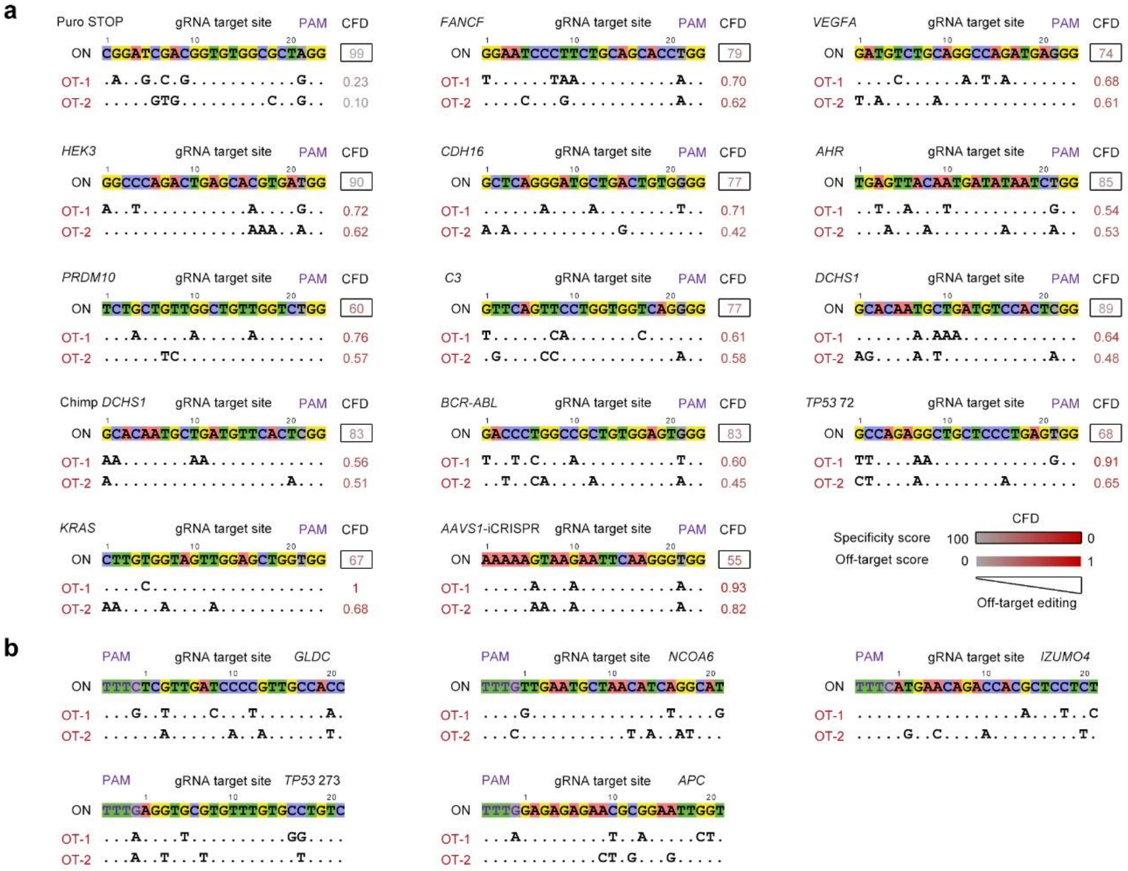

Supplementary Figure 7: Predicted off-target sites. (**a**) Target site sequences of Cas9 SNIPE-gRNAs used in this study as well as their respective two off-targets with the highest cutting frequency determination (CFD) scores [44] are shown below the on-target sites. Identical bases are given by dots. The CFD specificity score is framed black. (**b**) Target site sequences of Cas12a SNIPE-gRNAs used in this study as well as their respective two off-targets with the lowest number of mismatches are shown below the on-target sites.

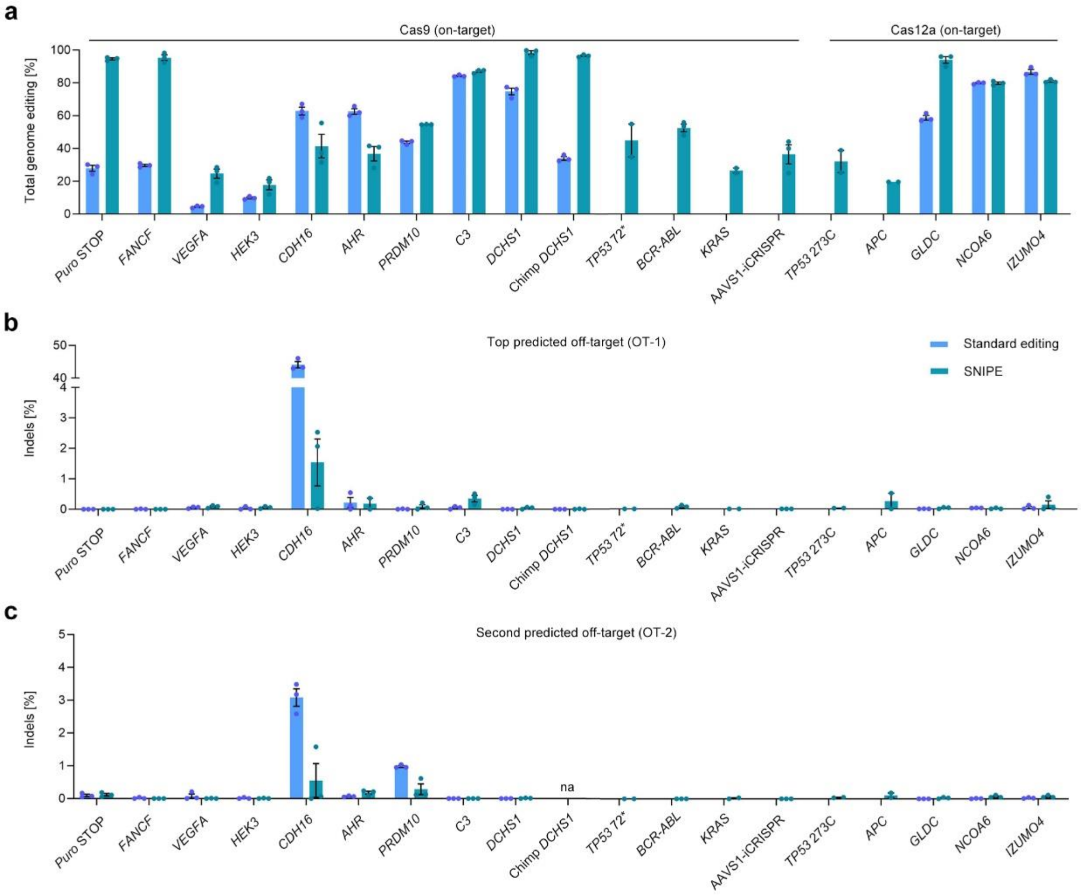

Supplementary Figure 8: Impact of SNIPE on off-target editing. (**a**) Total genome editing at on-target sites for standard editing (blue) and SNIPE (turquoise) used in this study. (**b**) Indels at the top predicted off-target sites for gRNAs from a. (**c**) Indels at the second predicted off-target sites for gRNAs from a. Standard editing was done using inducible Cas9 prime editing, Cas9 RNP with DNA donor, or Cpf1-Ultra RNP with DNA donor. SNIPE was done using Cas9 HiFi RNP or Cpf1-Ultra RNP after precision editing with prime editing or HDRobust. Editing was done in 409B2 human iPSCs, except for: chimp *DCHS1* (chimpanzee Sandra A iPSCs), *BCR-ABL* (K562), *KRAS* (HuCCT1), *TP53* 273C (RDES), and *APC* (HT29). We could not amplify the OT-2 locus for chimpanzee DCHS1 using combinations of four different primers. Independent biological replicates are depicted by dots (n = 2 or 3) and the error bars show the s.e.m.

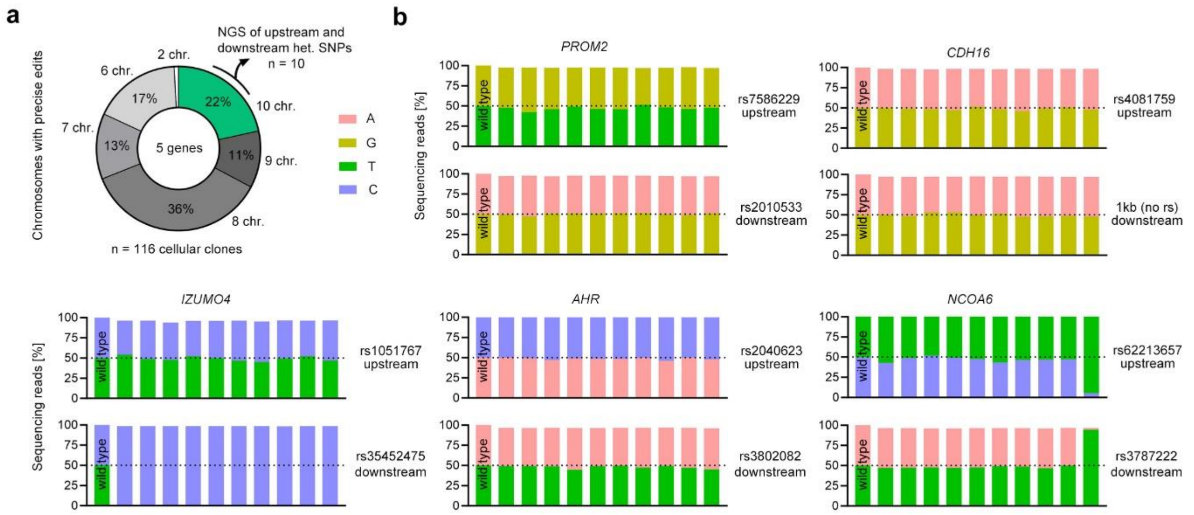

Supplementary Figure 9: Neighbouring SNP genotype of single cell-derived clones after multiplexed SNIPE. 409B2 iPrime iPSCs were edited for five genes by a combination of multiplexed prime editing, HDR based point mutations and SNIPE. (**a**) Percentage of precisely edited chromosomes for five genes in cellular clones (n = 116). Clones with precise edits on all targeted 10 chromosomes are highlighted in green. 10 deca-edited clones were further characterized by NGS of amplicons covering a normally heterozygous SNP upstream and downstream of the target loci (**b**), Genotypes of SNPs upstream and downstream of the target site for all five genes in ten cellular clones edited for all 10 chromosomes. The rsID numbers of the SNPs are stated.
